# Relationship between the Relaxation of Ionic Liquid Structural Motifs and That of the Shear Viscosity

**DOI:** 10.1021/acs.jpcb.1c03105

**Published:** 2021-06-07

**Authors:** Weththasinghage D. Amith, Juan C. Araque, Claudio J. Margulis

**Affiliations:** † Department of Chemistry, University of Iowa, Iowa City, Iowa 52242, United States; ‡ School of Engineering, Benedictine College, Atchison, Kansas 66002, United States

## Abstract

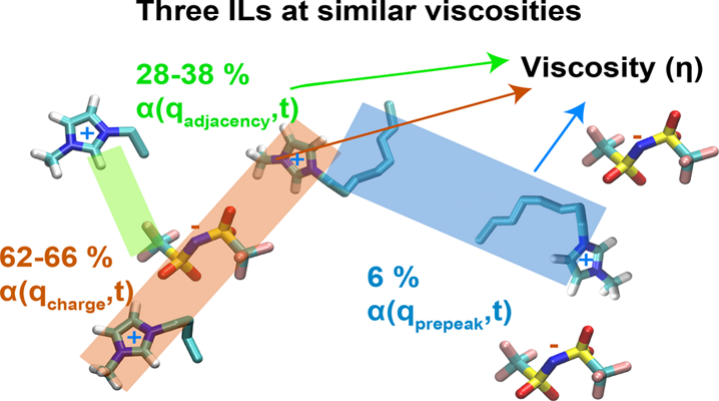

In a set of recent articles, we have highlighted that friction is highly inhomogeneous in a typical ionic liquid (IL) with charge networks that are stiff and charge-depleted regions that are soft. This has consequences not only for the dynamics of ILs but also for the transport properties of solutes dissolved in them. In this article, we explore whether the family of alkylimidazolium ILs coupled with bis(trifluoromethylsulfonyl)imide (with similar Coulombic interactions but different alkyl tails), when dynamically “equalized” by having a similar shear viscosity, display *q*-dependent structural relaxation time scales that are the same across the family. Our results show that this is not the case, and in fact, the relaxation of in-network charge alternation appears to be significantly affected by the presence of separate polar and apolar domains. However, we also find that if one was to assign weight factors to the relaxation of the structural motifs, charge alternation always contributes about the same amount (between 62.1 and 66.3%) across systems to the running integral of the stress tensor correlation function from which the shear viscosity is derived. Adjacency correlations between positive and negative moieties also contribute an identical amount if a prepeak is not present (about 38%) and a slightly smaller amount (about 28%) when intermediate range order exists. The prepeak only contributes about 6% to viscoelastic relaxation, highlighting that the dynamics of the smaller scale motifs is the most important.

## Introduction

**Figure 1:**
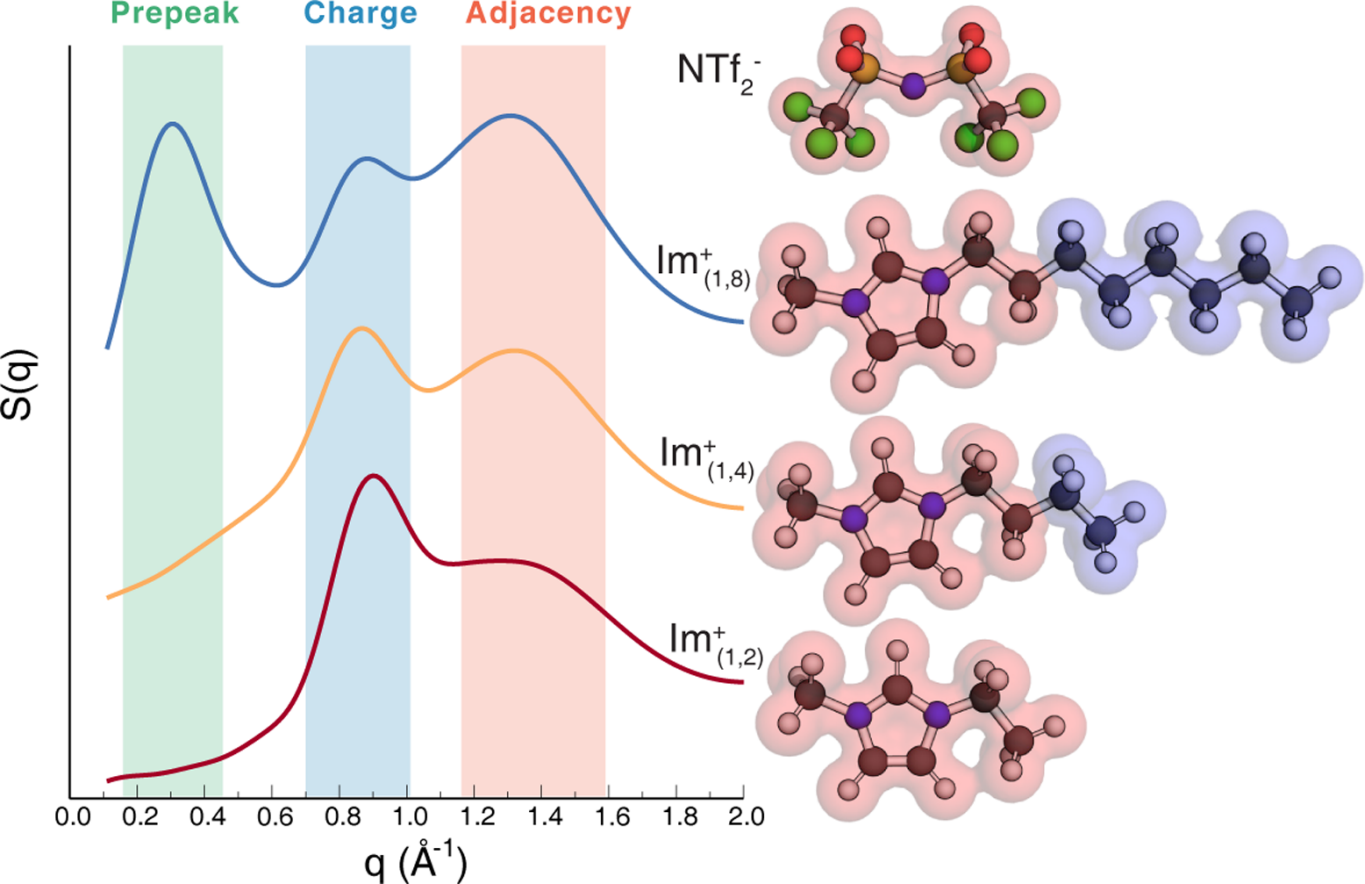
Static structure function *S*(*q*) for Im_1,2_^+^/NTf_2_^–^, Im_1,4_^+^/NTf_2_^–^, and Im_1,8_^+^/NTf_2_^–^ under the approximate isoviscosity condition μ ≈ 8 cP. In the figure, we also show our definition of cation head and anion components in red and tail components in blue. Notice that for Im_1,2_^+^, the full cation is considered the cationic head.

**Figure 2:**
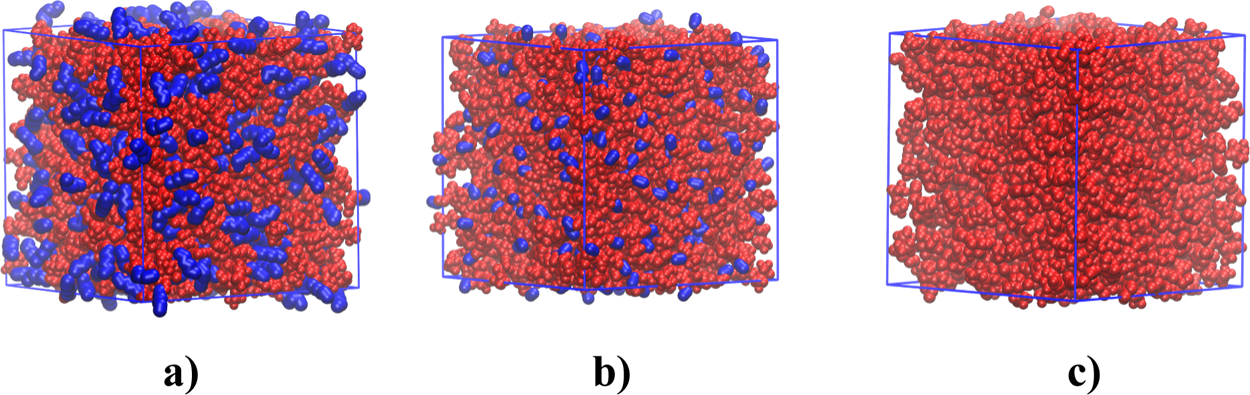
Snapshots of (a) Im_1,8_^+^/NTf_2_^–^, (b) Im_1,4_^+^/NTf_2_^–^, and (c) Im_1,2_^+^/NTf_2_^–^. Charge heads and anions are in red and apolar components in blue (the color code matches that in Figure 1).

In all real-life applications of ionic liquids (ILs),^1,2^ including in the fields of tribology,^3−7^ batteries and capacitors,^1,8,9^ heat transfer,^10,11^ separations,^12^ and even space exploration,^13^ an important property to consider is their shear viscosity. While many other characteristics of ILs may make them very advantageous, their viscosity at a given operational temperature can ultimately dictate whether a process is economically feasible. In this article, we continue our exploration of the link between structural dynamics,^14−26^ as described by the partial subcomponents of the dynamic structure function [*S*(*q*,*t*)], and transport properties, in particular shear relaxation. We focus on three prototypical liquids from the same family depicted in Figure 1, namely, 1-methyl-3-octylimidazolium bis(trifluoromethylsulfonyl)imide (Im_1,8_^+^/NTf_2_^−^), 1-butyl-3-methylimidazolium bis(trifluoromethylsulfonyl)imide (Im_1,4_^+^/NTf_2_^−^), and 1-ethyl-3-methylimidazolium bis(trifluoromethylsulfonyl)imide (Im_1,2_^+^/NTf_2_^−^) at temperatures such that their viscosity is approximately 8 cP. We work approximately at the same viscosity instead of at the same temperature in an attempt to “equalize” their dynamics and frictional characteristics. The reader is encouraged to look at ref (24) and in particular its Figure 2 where the isoviscosity condition is discussed in the context of Maroncelli’s real versus ideal friction ratios;^27^ these authors as well as others^28,29^ have discussed how the rotational and translational dynamics of solutes deviate from hydrodynamics predictions in ILs based on their size and charge characteristics. In ref (24), we found that Maroncelli’s deviation trends are still valid but less pronounced when ILs are considered at isoviscosity instead of at the same temperature where their viscosities and frictional characteristics can differ significantly. Our study at an approximate isoviscosity condition is in contrast, for example, to results presented in ref (30), where the structural dynamics of Im_1,8_^+^/NTf_2_^–^ and Im_1,2_^+^/NTf_2_^–^ were studied at the same temperature and the viscosity of one of the systems was about twice that of the other.

In order to better understand the results we are about to present, we show in Figure 1 the static structure function *S*(*q*) for our three systems each at its own respective temperature consistent with the quasi-isoviscous condition. The computed viscosities are 7.9 cP for Im_1,2_^+^/NTf_2_^–^, 7.7 cP for Im_1,4_^+^/NTf_2_^–^, and 8.8 cP for Im_1,8_^+^/NTf_2_^–^ at 354.4, 363.3 and 377.4 K, respectively, and errors are displayed in Figure S1. As is to be expected, the structure function can be divided into three *q*-regions associated with adjacency correlations, charge alternation, and, when present, intermediate range order as evidenced by a prepeak or first sharp diffraction peak.^19,20,31−43^ Only Im_1,8_^+^/NTf_2_^–^ displays the signature of nanostructural heterogeneity associated with a prepeak. The origin of the prepeak is well understood from prior studies and stems from the alternation of charge networks and apolar domains as can be gleaned in 3D from Figure 2a. The reader can see how these domains do not really form for the shorter alkyl tail liquids in Figure 2b,c; such behavior has been observed for many ILs before.^3,44−49^

## Computational Methods

Simulation and analysis details follow a similar protocol to that in a prior study.^26^ All simulation data for Im_1,8_^+^/NTf_2_^–^ are from refs (24) and (26). Initial steps (equilibration and constant energy simulations) for Im_1,4_^+^/NTf_2_^–^ and Im_1,2_^+^/NTf_2_^–^ are also from ref (24); therefore, we only provide a brief recapitulation of these steps here. In all cases, 512 ion pairs were used; these were initially energy-minimized and later equilibrated by scaling charges (first at 0% and then at 10%) in the constant temperature and pressure (*NPT*) ensemble for 0.5 ns. Systems were then subjected to simulated annealing in the same ensemble from target temperature up to 700 K and back to target temperature at full charge for 5 ns. Following these steps, 10 ns at constant volume and energy (*NVE* ensemble) were simulated to compute *S*(*q*,*t*); subsequent viscosity calculations (*vide infra*) were carried out in the constant volume and temperature (*NVT*) ensemble at the average temperature derived from the aforementioned NVE runs.

Potential parameters are from Canongia-Lopes and Pádua^50,51^ and the optimized potentials for liquid simulations all-atoms^52^ force fields and the Köddermann et. al. modifications to Lennard-Jones parameters only.^53^ Final configurations of pre-equilibrated *NVE* runs for Im_1,2_^+^/NTf_2_^–^ and Im_1,4_^+^/NTf_2_^–^ from a previous study^24^ were used as input for a series of constant temperature *NVT* runs from which the stress tensor auto-correlation function was computed. From these trajectories, the viscosity was computed as described in ref (54). To generate our *NVT* trajectories computed in double precision, we used GROMACS version (4.5.5).^55,56^ Eighty independent *NVT* trajectories (7 ns each run) were needed to converge the viscosity of Im_1,2_^+^/NTf_2_^–^ and Im_1,4_^+^/NTf_2_^–^. For our *NVT* runs, we used the Nosé-Hoover^57,58^ thermostat with a 0.5 ps time constant and the velocity-Verlet^59^ MD integrator. The cutoff for all the non-bonded interactions was considered to be 1.5 nm and electrostatic interactions were computed using the Particle-mesh Ewald method^60,61^ with 0.08 nm Fourier spacing and a sixth order interpolation.

The dynamic structure function is defined as^26^
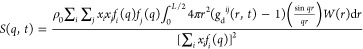
1where *g*_d_^*ij*^(*r*,*t*) is the distinct van Hove correlation function computed with periodic boundary conditions from our in-house modified version of LiquiLib toolbox normalized so that at time 0 it becomes the pair distribution function *g*^ij^(*r*) and at long time it goes to 1.^62^ In Equation (1) *x*_*i*_, *x*_*j*_ are the corresponding atomic mol fractions, *f*_*i*_(*q*), *f*_*j*_(*q*) are the X-ray atomic form factors, and *W*(*r*) is a Lorch function. Just like the structure function *S*(*q*), the dynamic structure function can also be partitioned into convenient additive subcomponents^19,20,31−43^ that provide useful information about the correlation of specific subgroups of atoms in the liquid; in this work, we focus mostly on its cationic head–anion subcomponent, *S*^H–A^(*q*,*t*). In order to compute (i) the normalized (going to 1 at a long time) running time integral of the stress tensor auto-correlation function
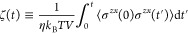
2where the shear viscosity η is defined from the Green Kubo relation^63^ as
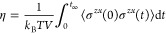
3and (ii) the normalized running time integral of the head–anion subcomponent of the dynamic structure function squared
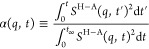
4cutoff values corresponding to *t*_∞_ needed to be defined as in the protocol described in ref (54). For Im_1,2_^+^/NTf_2_^–^ and Im_1,4_^+^/NTf_2_^–^, *t*_∞_ was set to 675 ps and 900 ps respectively; as described in ref (26), *t*_∞_ for Im_1,8_^+^/NTf_2_^–^ was set to 984 ps, except at *q*_prepeak_ where it was set to 2500 ps. The idea behind the study of α(*q*,*t*), which reflects the time relaxation of IL structural motifs, is that structural relaxation and viscoelastic relaxation are necessarily linked. Mode coupling theory combined with hypotheses put forth by Yamaguchi^26,30,63−71^ imply that ∫_0_^*t*^*S*(*q*,*t*^′^)^2^d*t*^′^ evaluated at a specific q value and ∫_0_^*t*^⟨σ^*zx*^(0)σ^*zx*^(*t*^′^)⟩d*t*^′^ are related quantities. When normalized as in the definitions of ζ(*t*) and α(*q*,*t*), the viscoelastic relaxation and the structural relaxation of IL motifs can be directly contrasted.

## Results and Discussion

In a prior study,^26^ we linked the shear relaxation dynamics of Im_1,8_^+^/NTf_2_^–^ to the loss of memory about the location of charges within (or along as opposed to across) charge networks. Charge networks are the stiff^19−26,72^ part of the liquid and it is reasonable to imagine that such loss of memory results from the motion of nearby charged species that are strongly coupled electrostatically and that produce significant shear stress on each other. Instead, the slower across networks loss of memory associated with the prepeak requires much larger scale liquid rearrangements. For example, in the prepeak decorrelation regime, loss of memory implies apolar regions flooding the original location of charge networks, charged components that were originally part of one charge network crossing or pinching an apolar domain in order to swap charge strands, or other rearrangements that are collective and on a lengthscale significantly larger than the ions.

**Figure 3:**
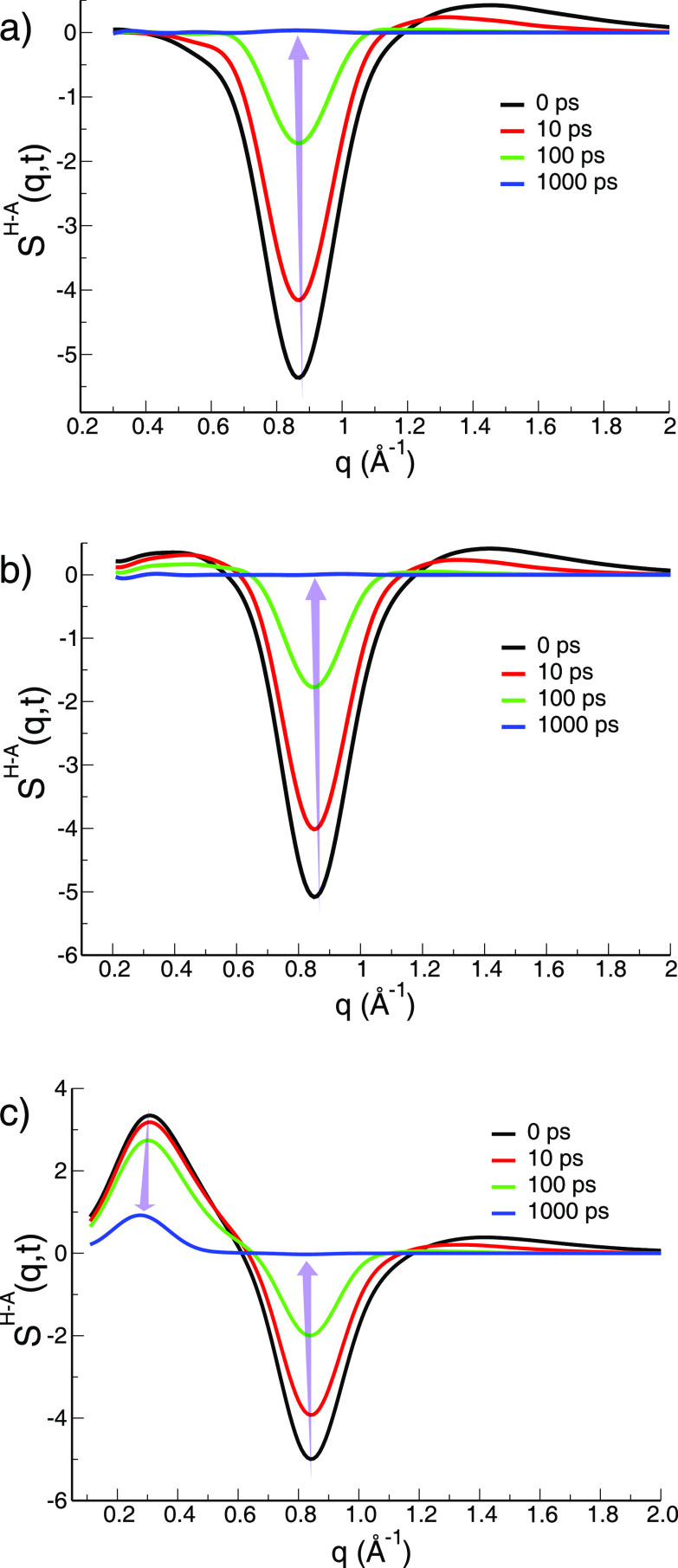
Time evolution of *S*^H–A^(*q*,*t*) for (a) Im_1,2_^+^/NTf_2_^–^, (b) Im_1,4_^+^/NTf_2_^–^, and (c) Im_1,8_^+^/NTf_2_^–^.

Since the dynamics of the charge network appears to be so important to the viscosity,^19−21,23−26,30,64^ we should remind the reader why a charge alternation peak is often absent in the X-ray and neutron scattering *S*(*q*) or *S*(*q*,*t*).^20,36^ Whether the charge alternation feature shows up at all in *S*(*q*) or not depends completely on the contrast for the technique and not so much on the actual topology of the liquid.^20^ This is because the overall *S*(*q*) can be partitioned into subcomponents^20,31,33^ that in the charge alternation regime frequently cancel.^20,36,40^ Specifically, cationic head–head correlations and anion–anion correlations show as peaks in this *q*-region whereas cationic head–anion correlations show as what we have termed as an antipeak (see negative going peaks in Figure 3a–c at 0 ps in the regime *q* ≈ 0.85 Å^–1^).^40^ This antipeak is often of large enough intensity to cancel the sum of cationic head–head and anion–anion peaks. The very interesting characteristics of the cationic head–anion subcomponent of *S*(*q*) are universal across ILs and are thoroughly discussed in ref (40).

In this article, we focus on the dynamics of *S*^H–A^(*q*,*t*) as displayed for our three liquids under the approximate isoviscosity condition in Figure 3.^19,20,31−43^ The reason for this is that this subcomponent has the most intuitive physical correlation with the three structural motifs of ILs. We have shown in prior studies^19,20,31−43^ that when there is an alternation between classes of species (in ILs, these can be subspecies, like tails and heads), the same-type correlations appear as peaks and the opposite-type correlations as antipeaks. In the prepeak region, when a prepeak exists, *S*^H–A^(*q*,*t*) always appears as a prominent peak because both cation heads and anions are considered part of the same polar species which alternates with the apolar domains (see the lowest *q*-peak at *q* ≈ 0.3 Å^–1^ in Figure 3c at time 0). Instead, in the charge alternation regime, *S*^H–A^(*q*,*t*) always shows as an antipeak (see Figure 3a–c at time 0 in the charge alternation region (*q* ≈ 0.85 Å^–1^)). This is because in this *q*-region, cationic heads and anions are considered different species that alternate due to the value of their charge. This antipeak signifies that at the typical distance we expect to find cation heads from other cation heads, there is a depletion of probability of finding anions and vice versa. *S*^H–A^(*q*) also has a clear physical meaning in the adjacency regime (*q* ≈ 1.4 Å^–1^) as it highlights the most common nearest-neighbor correlations in a salt, which are those between oppositely charged species. It is therefore clear that the dynamics of *S*^H–A^(*q*,*t*) in the three different *q* regimes (peak at *q*_prepeak_, antipeak at *q*_charge_, and peak at *q*_adjacency_) is directly related to the decay of correlations of the prototypical IL motifs we all can easily visualize and understand.

If we want to relate our results to the viscosity, the key quantity to study is not *S*^H–A^(*q*,*t*) but instead the normalized running integral of its square value, α(*q*,*t*), as defined in the methods section; this stems from mode coupling theoretical considerations that have been described previously.^26,30,63−71^ To be clear about why we focus on this subcomponent of the dynamic structure function, Figures S2–S4 show that the time evolution of the three structural motifs in α(*q*,*t*) is qualitatively very similar to that of the overall (∫_0_^*t*^*S*(*q*,*t*′)^2^ d*t*′/∫_0_^*t*_∞_^*S*(*q*,*t*)^2^ d*t*) in the same three *q* regimes. However, the interpretation of results is much simpler for α(*q*,*t*) as its meaning is clear and not polluted with contributions from a milliard other more difficult to understand correlations.

**Figure 4:**
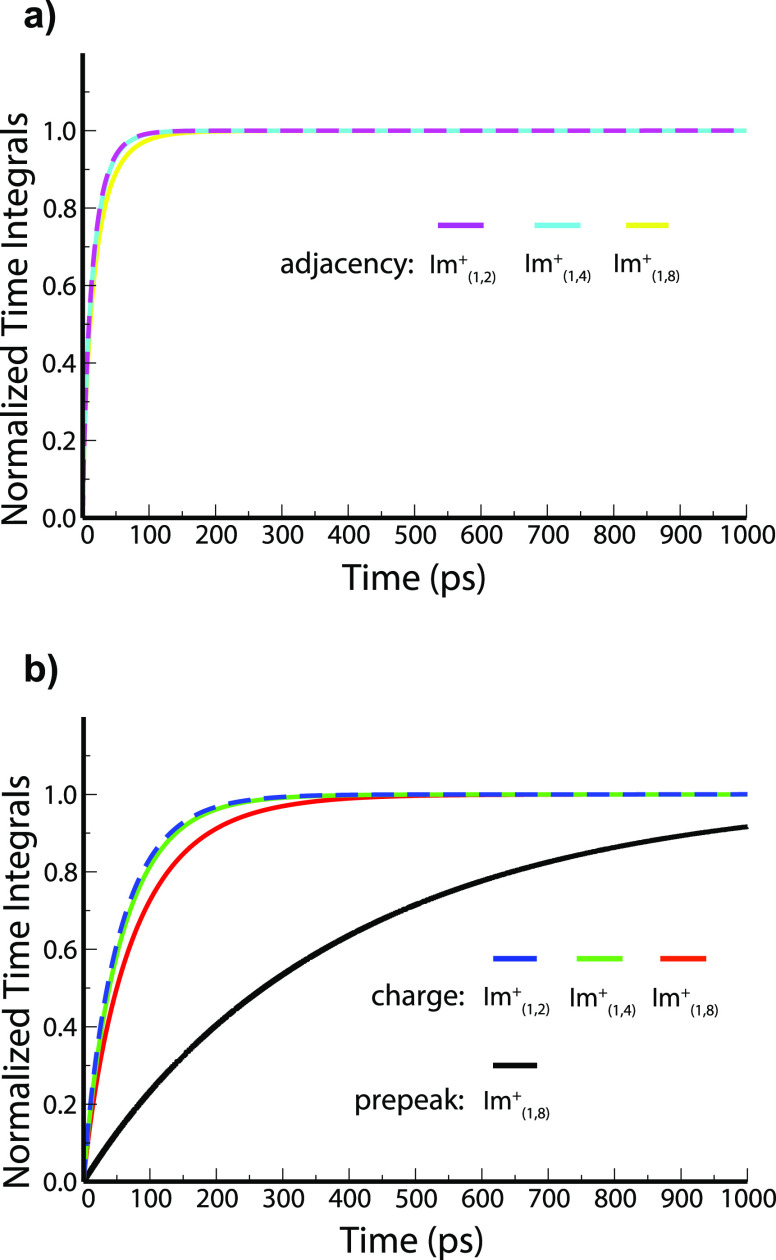
α(*q*,*t*) for Im_1,2_^+^/NTf_2_^–^, Im_1,4_^+^/NTf_2_^–^, and Im_1,8_^+^/NTf_2_^–^ at different *q* values. For Im_1,8_^+^/NTf_2_^–^, *q*_prepeak_ = 0.3 Å^–1^, *q*_charge_ = 0.85 Å^–1^, and *q*_adjacency_ = 1.4 Å^–1^, for Im_1,4_^+^/NTf_2_^–^, *q*_charge_ = 0.85 Å^–1^ and *q*_adjacency_ = 1.4 Å^–1^, and for Im_1,2_^+^/NTf_2_^–^, *q*_charge_ = 0.86 Å^–1^ and *q*_adjacency_ = 1.4 Å^–1^. For clarity, (a) shows adjacency curves and (b) charge alternation and prepeak curves.

Figure 4a,b shows for our three systems α(*q*,*t*) at the *q*-values corresponding to the structural motifs. We see that the relaxation of adjacency correlations is almost identical for Im_1,2_^+^/NTf_2_^–^ and Im_1,4_^+^/NTf_2_^–^ and only slightly slower for Im_1,8_^+^/NTf_2_^–^; these are the fastest relevant structural correlations in ILs corresponding to nearest-neighbor dynamics. When it comes to the relaxation of the charge alternation feature, we see that it is slightly slower for Im_1,4_^+^/NTf_2_^–^ than for Im_1,2_^+^/NTf_2_^–^ even though the latter is a little more viscous in our study. The effect of larger alkyl tails becomes even more apparent when we look at Im_1,8_^+^/NTf_2_^–^, for which the relaxation of the charge alternation motif is the slowest across the three ILs. We remind the reader that the relaxation of the charge alternation peak is related to charge blurring within networks or strands ubiquitous across all ILs. These results are consistent with the observations by Yamaguchi^64^ that with an increase in length of the alkyl tail, the relaxation time of the charge alternation feature is more significantly affected than the actual value of the viscosity. Only Im_1,8_^+^/NTf_2_^–^ has a prepeak, and it is clear from Figure 4b that the relaxation of the polar–apolar alternation motif is by far the slowest of all features.

**Figure 5:**
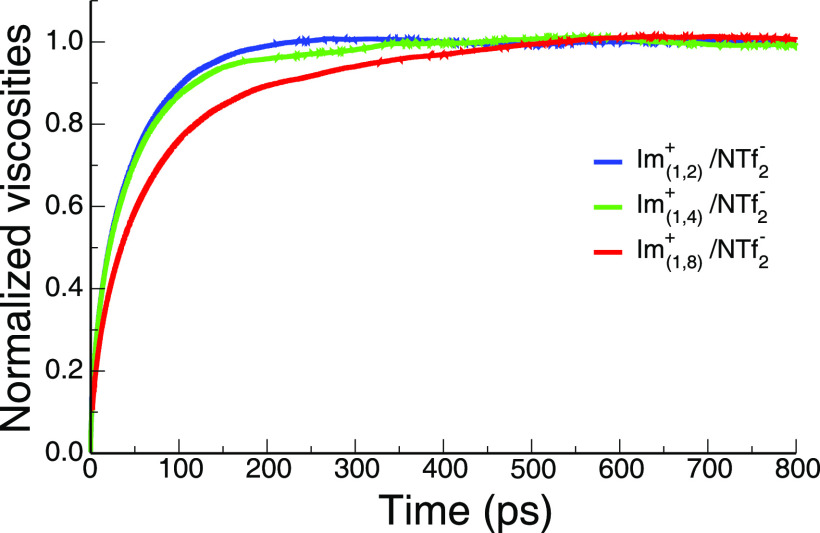
ζ(*t*) for Im_1,2_^+^/NTf_2_^–^, Im_1,4_^+^/NTf_2_^–^, and Im_1,8_^+^/NTf_2_^–^.

The reader is asked to notice from Figure 5 that whereas all three liquids are at a similar viscosity, the normalized time integrals of the stress tensor auto-correlation ζ(*t*), defined in the methods section, follow the same trend as the charge alternation motif relaxation in Figure 4b. The relaxation is fastest for Im_1,2_^+^/NTf_2_^–^, intermediate for Im_1,4_^+^/NTf_2_^–^, and slowest for Im_1,8_^+^/NTf_2_^–^, with the difference between Im_1,8_^+^/NTf_2_^–^ and Im_1,4_^+^/NTf_2_^–^ being much larger than between Im_1,2_^+^/NTf_2_^–^ and Im_1,4_^+^/NTf_2_^–^ (biexponential time constants for the relaxation of ζ(*t*) are given in Table S1).^73^

**Figure 6:**
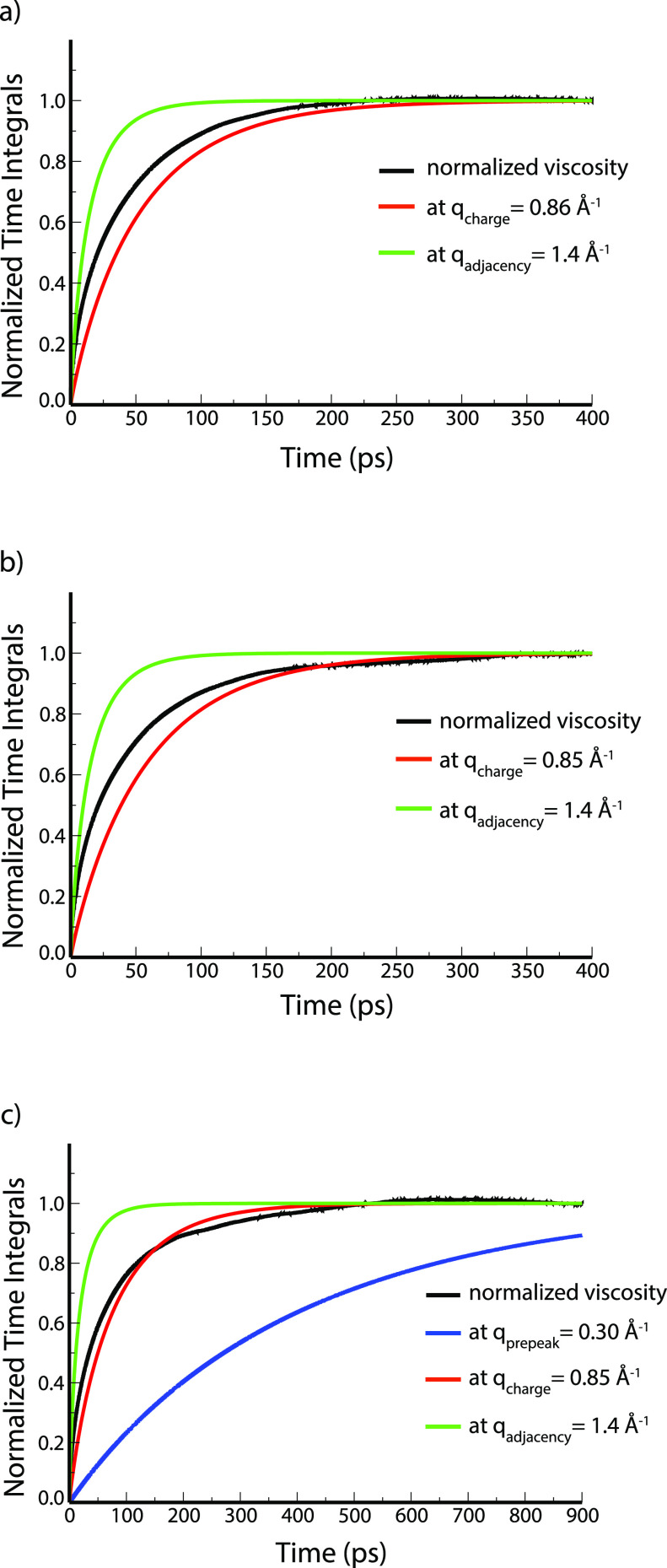
ζ(*t*) compared to α(*q*,*t*) at the *q* values of the structural motifs for Im_1,2_^+^/NTf_2_^–^ (a), Im_1,4_^+^/NTf_2_^–^ (b), and Im_1,8_^+^/NTf_2_^–^ (c) (a similar plot in the case of Im_1,8_^+^/NTf_2_^–^ was already published in ref (26)).

Figure 6 compares the relaxation of the motifs as seen from the time evolution of α(*q*,*t*) with that of ζ(*t*). The first obvious conclusion from these figures is that shear relaxation falls somewhere between that of the fastest and slowest relevant structural motif in the liquid. In Im_1,2_^+^/NTf_2_^–^ and Im_1,4_^+^/NTf_2_^–^, it falls between that of adjacency and charge alternation motifs and in Im_1,8_^+^/NTf_2_^–^ between that of adjacency and polar–apolar alternation motifs. We described in a prior publication,^26^ that for Im_1,8_^+^/NTf_2_^–^, the relaxation of α(*q*,*t*) for the charge alternation motif is qualitatively quite similar to that of ζ(*t*) and this can be gleaned from Figure 6c.^26^ However, a more mathematically formal comparison between the dynamics of the structural motifs and that of the shear relaxation will become quite revealing.

**Figure 7:**
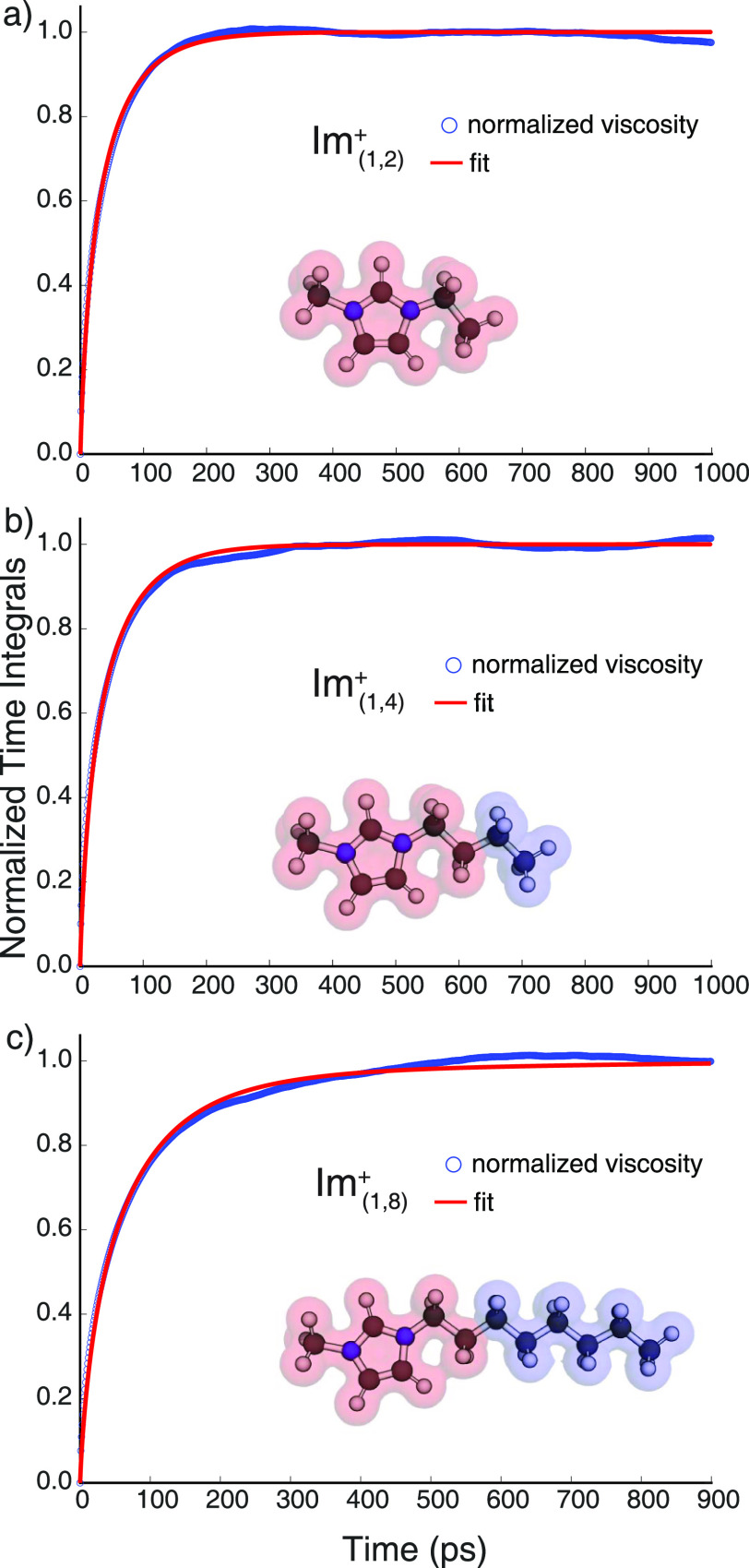
For Im_1,2_^+^/NTf_2_^–^ (a), Im_1,4_^+^/NTf_2_^–^ (b), and Im_1,8_^+^/NTf_2_^–^ (c), fits of the coefficients in the expression ζ(*t*) = *c*_adjacency_ × α(*q*_adjacency_, *t*) + *c*_charge_ × α(*q*_charge_, *t*) + *c*_polarity_ × α(*q*_polarity_, *t*). For Im_1,2_^+^/NTf_2_^–^ and Im_1,4_^+^/NTf_2_^–^, the last term in the sum is not considered.

Two key questions need to be answered; can we assign weights to α(*q*,*t*) at the three (two) relevant *q*-values corresponding to the motifs so that a linear combination faithfully recovers ζ(*t*)? If so, how do these weights compare across ILs? The answers to these questions are quite remarkable as can be gleaned from Figure 7 and Table 1. We fit the equation ζ(*t*) = *c*_adjacency_ × α(*q*_adjacency_, *t*) + *c*_charge_ × α(*q*_charge_, *t*) + *c*_polarity_ × α(*q*_polarity_, *t*) (or in the case of Im_1,2_^+^/NTf_2_^–^ and Im_1,4_^+^/NTf_2_^–^ where there is no prepeak ζ(*t*) = *c*_adjacency_ × α(*q*_adjacency_, *t*) + *c*_charge_ × α(*q*_charge_, *t*)) with the additional constraint that *c*_adjacency_ + *c*_charge_ + *c*_polarity_ = 1 (or *c*_adjacency_ + *c*_charge_ = 1) using the fmincon algorithm as coded in MATLAB.^74^ Even though, visually, α(*q*_charge_, *t*) in Figure 6 appears quite similar to ζ(*t*) for Im_1,8_^+^/NTf_2_^–^ and less so for other ILs, in reality the weight *c*_charge_ is similar across the family. Specifically, the charge network dynamics appears to contribute about 62% in the case of ILs without apolar domains and about 66% for Im_1,8_^+^/NTf_2_^–^ to the dynamics of the stress tensor relaxation. In fact, for Im_1,2_^+^/NTf_2_^–^ and Im_1,4_^+^/NTf_2_^–^, adjacency correlations also contribute the same amount to ζ(*t*), about 38%. Even in the case of Im_1,8_^+^/NTf_2_^–^, the contribution of adjacency correlations is still similar (about 28%) because *c*_polarity_ only contributes about 6% to ζ(*t*). However, even this small contribution from the prepeak may be important since α(*q*_polarity_, *t*) relaxes on such a longer time scale. For completion, we ask the reader to see Figure S5 where for the case of Im_1,8_^+^/NTf_2_^–^, we plot α(*q*,*t*) evaluated at *q*_charge_ together with some of its atomic pair subcomponents. We see that the dynamics of the subcomponents is essentially the same as that of α(*q*,*t*), implying that the results will be more or less generic for neutron or X-ray scattering and no specific pair of interactions with large X-ray or neutron weights will skew the results.

**Table 1:** Weights of α(*q*,*t*) at *q*_adjacency_ and *q*_charge_ and When Applicable *q*_polarity_ in Fits of ζ(*t*)^a^

IL	*c*_adjacency_	*c*_charge_	*c*_polarity_
Im_1,8_^+^/NTf_2_^–^	0.279	0.663	0.058
Im_1,4_^+^/NTf_2_^–^	0.376	0.624	n/a
Im_1,2_^+^/NTf_2_^–^	0.379	0.621	n/a

a ζ(*t*) = *c*_adjacency_ × α(*q*_adjacency_, *t*) + *c*_charge_ × α(*q*_charge_, *t*) + *c*_polarity_ × α(*q*_polarity_, *t*) with the constraint *c*_adjacency_ + *c*_charge_ + *c*_polarity_ = 1. In the cases of Im_1,2_^+^ and Im_1,4_^+^, there is no intermediate range order and therefore we do not consider *c*_polarity_ and α(*q*_polarity_, *t*).

We interpret our results to mean that the relaxation of shear viscosity in these systems is dominated primarily by the blurring of the charge network and secondarily by the shorter range interactions of adjacent ions. Whereas the relaxation of the charge alternation feature is different for ILs with and without apolar domains (as can be seen from Figure 4b, even at isoviscosity, it is slower when there is intermediate range order), the contribution of it to ζ(*t*) is similar across liquids; if charge blurring occurs on a longer time scale, so will the relaxation of ζ(*t*).

## Conclusions

When we attempt to equalize the dynamics of a family of ILs with very similar charge interactions but different apolar components by studying them at temperatures that make their viscosity similar, we find that adjacency correlations relax fast and on a similar time scale across systems. Charge alternation dynamics is very similar for the ILs without a prepeak but somewhat slower when a prepeak is present. The dynamics of the prepeak is very slow compared to all other relevant structural relaxations. The relaxation of the three structural motifs (two when there are no apolar domains) can be linked to the viscoelastic relaxation. In trying to fit the relaxation of ζ(*t*), if each of the structural motifs is given a weight, we find that the weight for charge alternation is dominant and for practical purposes similar across ILs. This result is quite remarkable and deserves further scrutiny for other ILs and at other isoviscosity conditions. The contribution of adjacency correlations is also very significant and the same for ILs without a prepeak but slightly smaller when a prepeak is present. This may indicate that the viscoelastic relaxation in these systems could be dominated by first- and second-neighbor ion interactions as adjacency correlations are between neighbors and charge alternation correlations are those between second neighbors that give rise to IL charge networks. The prepeak contributes little because its relaxation is associated with the larger scale rearrangements of the polar and apolar motifs which occur on significantly slower time scales when compared to ζ(*t*).

## Supporting Information Available

Running integral of the Green–Kubo expression for viscosity including error bars; a comparison between ∫_0_^*t*^*S*(*q*,*t*′)^2^ d*t*′/∫_0_^*t*_∞_^*S*(*q*,*t*)^2^ d*t* and α(*q*,*t*) at the *q*-regions associated with the structural motifs; for Im_1,8_^+^/NTf_2_^–^ a comparison between α(*q*,*t*) and some of its partial subcomponents; parameters to fit ζ(*t*) including bi-exponential time constants (PDF)
