# Supplementary material for: Relationship between the Relaxation of Ionic Liquid Structural Motifs and That of the Shear Viscosity
Source: J Phys Chem B. 2021 Jun 7;125(23):6264–71. doi: 10.1021/acs.jpcb.1c03105 (PMC8279556; doi:10.1021/acs.jpcb.1c03105)
Supplement: Supplementary file 1 — jp1c03105_si_001.pdf [file jp1c03105_si_001.pdf]

Supporting Information for: Relationship between the Relaxation of Ionic Liquid Structural Motifs and That of the Shear Viscosity

Weththasinghage D. Amith,[†] Juan C. Araque,[‡] and Claudio J. Margulis[*,†]

†*Department of Chemistry, University of Iowa, Iowa City, Iowa 52242, United States*

‡*School of Engineering, Benedictine College, Atchison, Kansas 66002, United States*

E-mail: claudio-margulis@uiowa.edu

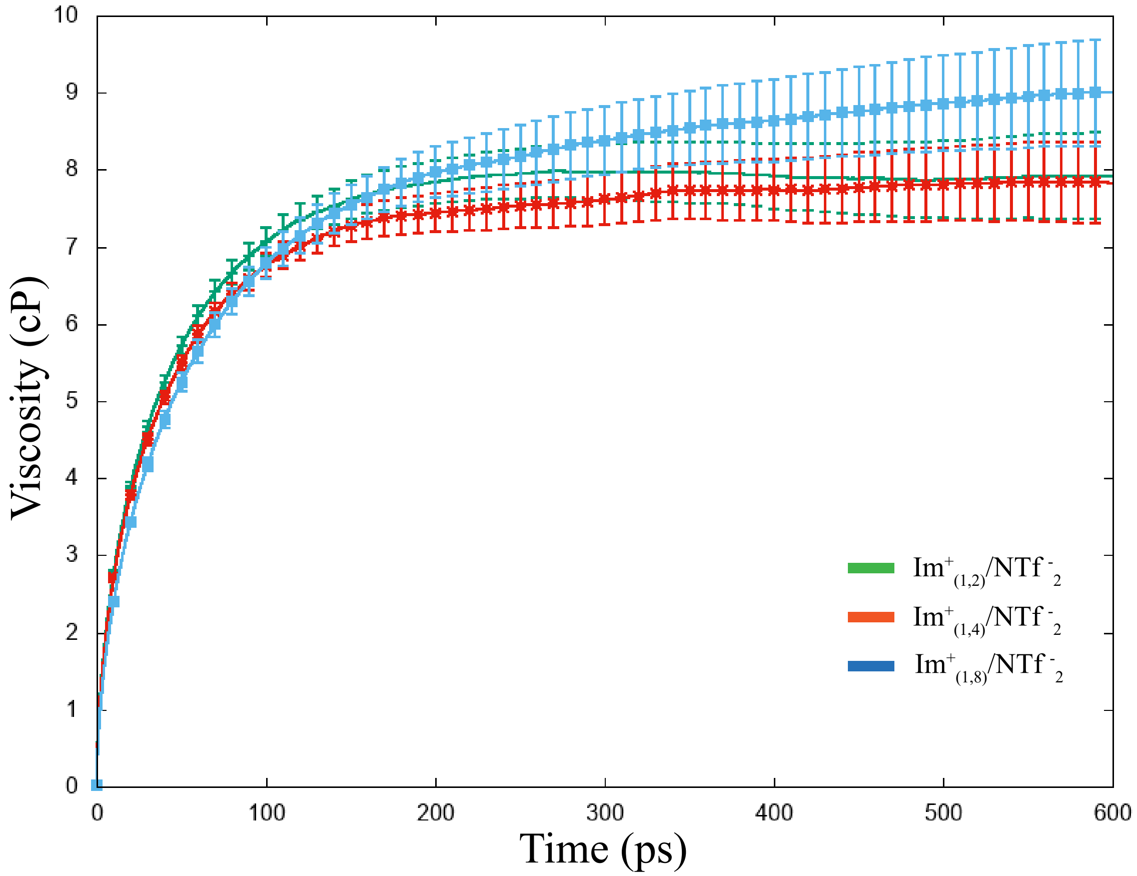

Figure S.1: The Green-Kubo expression $\frac{1}{k_B TV}\int_0^t \langle \sigma^{zx}(0)\sigma^{zx}(t')\rangle dt'$ and error bars at the 95 percent confidence level for $Im^+_{1,2}/NTf^-_2$, $Im^+_{1,4}/NTf^-_2$, and $Im^+_{1,8}/NTf^-_2$. The overlap in the error bars becomes even larger close to the corresponding t_∞ (data not show for clarity).

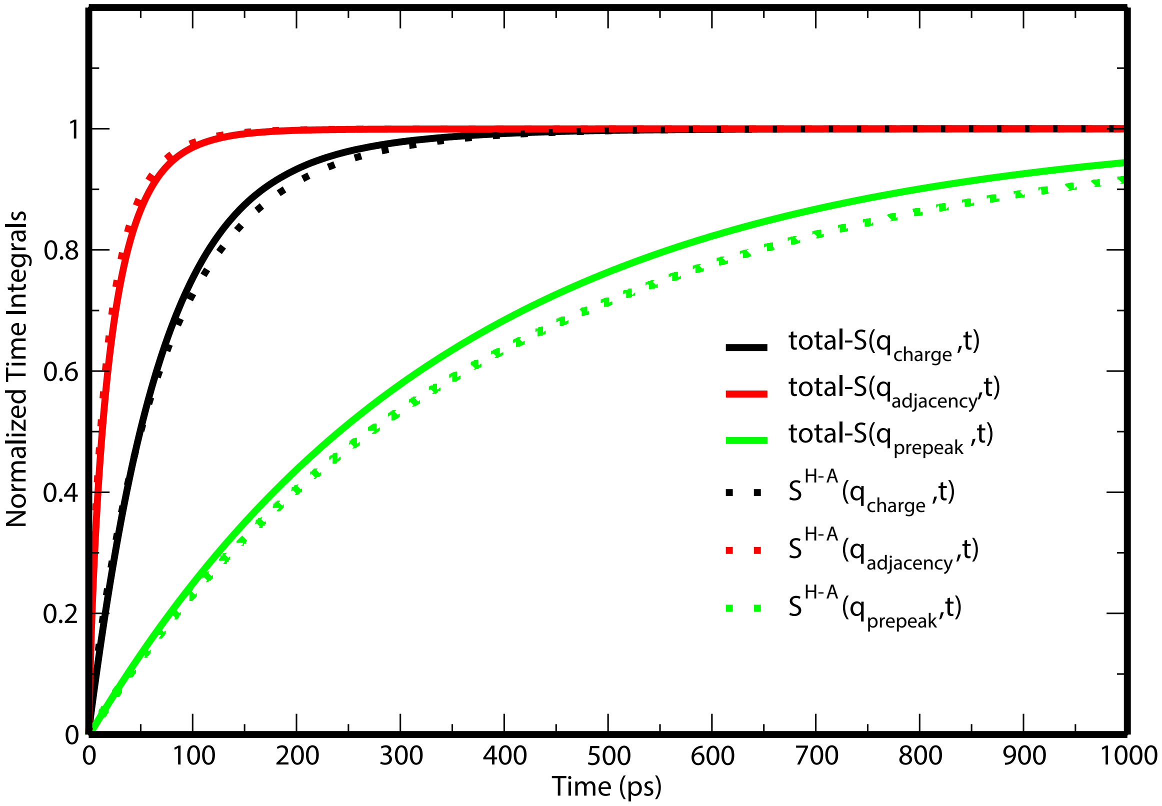

Figure S.2: For $Im^{+}_{1,8}/NTf^{-}_{2}$, a comparison between $\int_0^t S(q,t')^2\,dt' / \int_0^{t\infty} S(q,t)^2\,dt$ and $\alpha(q,t)$, at the q-regions associated with the structural motifs.

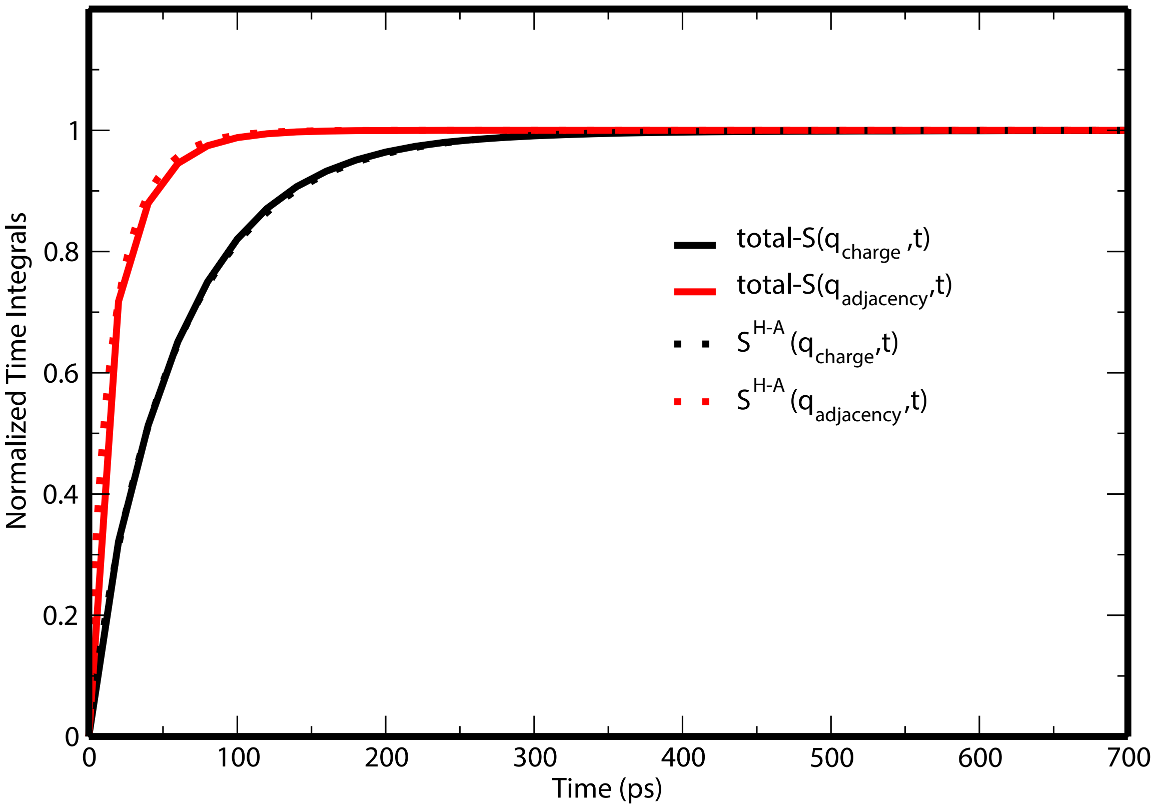

Figure S.3: For $Im^{+}_{1,4}/NTf^{-}_{2}$, a comparison between $\int_0^t S(q,t')^2\, dt' / \int_0^{t\infty} S(q,t)^2\, dt$ and $\alpha(q,t)$, at the q-regions associated with the structural motifs.

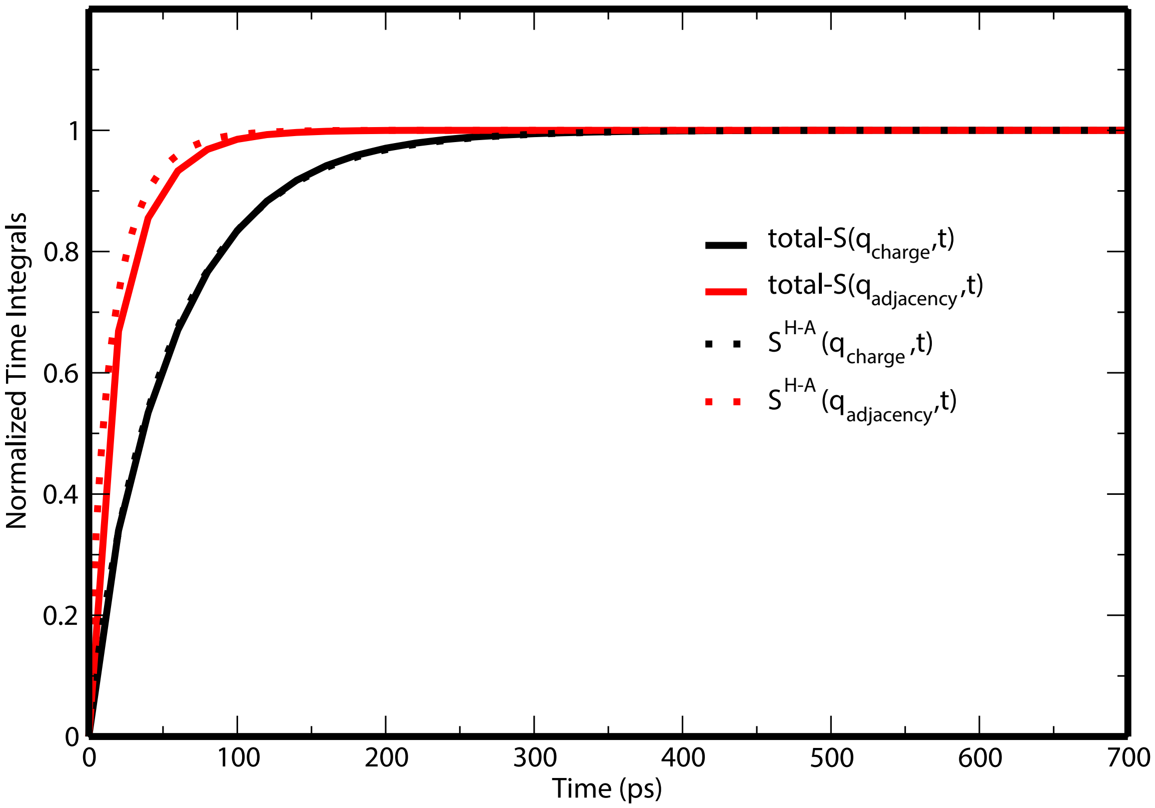

Figure S.4: For $Im^{+}_{1,2}/NTf^{-}_{2}$, a comparison between $\int_0^t S(q,t')^2\, dt' / \int_0^{t\infty} S(q,t)^2\, dt$ and $\alpha(q,t)$, at the q-regions associated with the structural motifs.

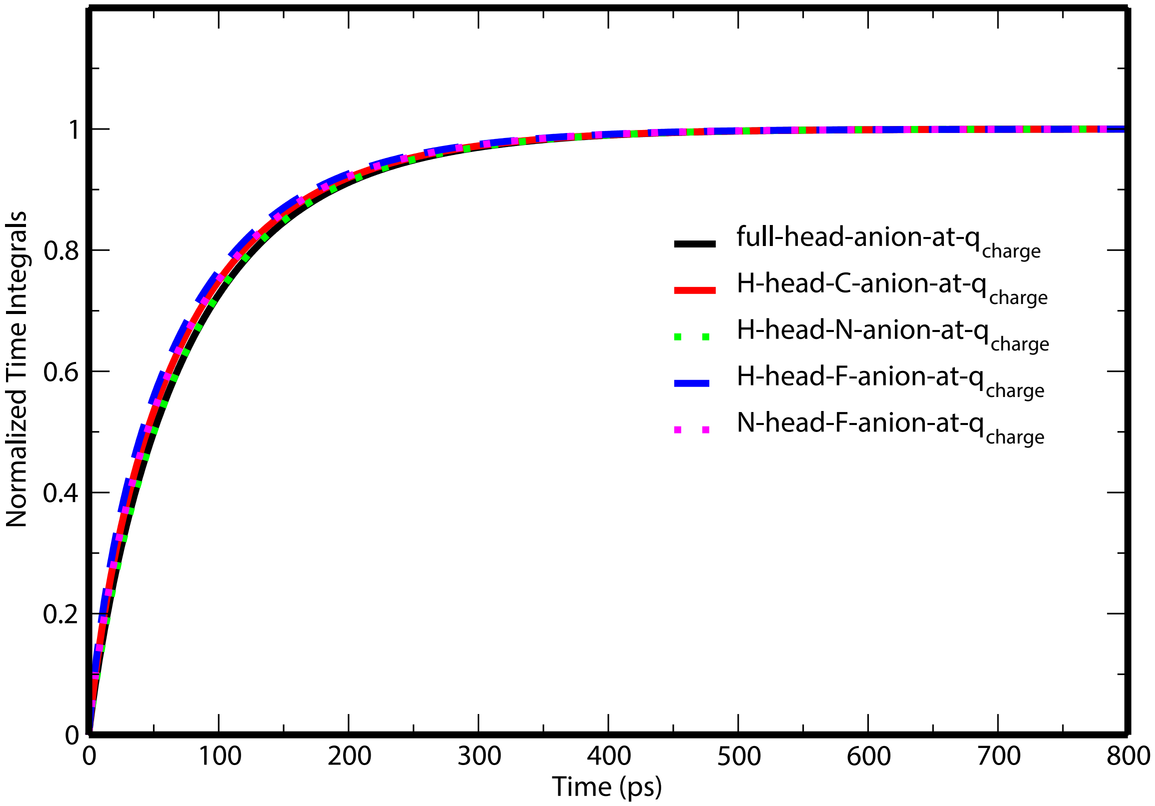

Figure S.5: For $Im^+_{1,8}/NTf^-_2$, α(q,t) compared with some of its partial atomic subcomponents.

Table S.1: Biexponential time constants (τ_1 and τ_2), as well as parameters A and α used to fit the relaxation of ζ(t) as defined in reference 1; computed viscosities for our three ILs. $\zeta(t) = A\alpha\tau_1(1 - e^{-t/\tau_1}) + A(1-\alpha)\tau_2(1 - e^{-t/\tau_2})$.

IL	Viscosity (cP)	A	α	τ_1 (ps)	τ_2 (ps)
$Im^{+}_{1,8}/NTf^{-}_{2}$	8.8 (at 377.4 K)	0.028491	0.135804	135.514	19.5938
$Im^{+}_{1,4}/NTf^{-}_{2}$	7.7 (at 363.3 K)	0.055910	0.175726	64.8021	7.86063
$Im^{+}_{1,2}/NTf^{-}_{2}$	7.9 (at 354.4 K)	0.081545	0.196336	47.9468	3.44041

References

(1) Zhang, Y.; Otani, A.; Maginn, E. J. Reliable Viscosity Calculation from Equilibrium Molecular Dynamics Simulations: A Time Decomposition Method. *Journal of Chemical Theory and Computation* **2015**, *11*, 3537–3546.
